# The Use of Phonetic Motor Invariants Can Improve Automatic Phoneme Discrimination

**DOI:** 10.1371/journal.pone.0024055

**Published:** 2011-09-01

**Authors:** Claudio Castellini, Leonardo Badino, Giorgio Metta, Giulio Sandini, Michele Tavella, Mirko Grimaldi, Luciano Fadiga

**Affiliations:** 1 LIRA-Lab, University of Genova, Genova, Italy; 2 Italian Institute of Technology, Genova, Italy; 3 Centro di Ricerca Interdisciplinare sul Linguaggio (CRIL), Salento University, Lecce, Italy; 4 DSBTA, University of Ferrara, Ferrara, Italy; The University of Western Ontario, Canada

## Abstract

We investigate the use of phonetic motor invariants (MIs), that is, recurring kinematic patterns of the human phonetic articulators, to improve automatic phoneme discrimination. Using a multi-subject database of synchronized speech and lips/tongue trajectories, we first identify MIs commonly associated with bilabial and dental consonants, and use them to simultaneously segment speech and motor signals. We then build a simple neural network-based regression schema (called Audio-Motor Map, AMM) mapping audio features of these segments to the corresponding MIs. Extensive experimental results show that 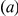 a small set of features extracted from the MIs, as originally gathered from articulatory sensors, are dramatically more effective than a large, state-of-the-art set of audio features, in automatically discriminating bilabials from dentals; 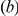 the same features, extracted from AMM-reconstructed MIs, are as effective as or better than the audio features, when testing across speakers and coarticulating phonemes; and dramatically better as noise is added to the speech signal. These results seem to support some of the claims of the motor theory of speech perception and add experimental evidence of the actual usefulness of MIs in the more general framework of automated speech recognition.

## Introduction

### Motivation

Automatic speech recognition (ASR) is the ability of a machine to convert human speech, coded as an audio signal, into words. Potential applications of ASR range from human-computer interfaces to informatics for the disabled to data mining in large speech corpora. While human beings show an excellent ability to understand one another's speech, independently of the speaker, the accent, the noise, etc., the robustness to speech variability of state-of-the-art ASR systems is still an active research topic.

Recent neuroscientific evidence indicates that the brain motor areas responsible for producing bilabial and dental phonemes are also involved in their perception, at least when speech is noisy. D'Ausilio et al. [1] show that in a noisy discrimination task of /b/ and /p/ versus /d/ and /t/, trans-cranial magnetic stimulation of the lips and tongue *motor areas* improves the *perception* of bilabials, and similarly, stimulation of the tongue favors dentals. This suggests that motor information may be paramount for speech understanding in humans.

Inspired by these findings, in this paper we investigate whether knowledge of speech production in humans, integrated into an automatic phone classifier, can improve the classification of /b/,/p/ versus /d/,/t/, in various conditions of noise and with different restrictions on the training set. To this end, we focus on the “artificial version” of the problem tackled in D'Ausilio et al.'s work, i.e., we perform the same classification task using computational models that combine auditory and motor information. For each consonant, a corresponding typical phonetic motor invariant (MI) is identified according to the basic physiology of speech; e.g., a fast opening (plosion) of the lips for /b/ and /p/ and of the tongue against the upper teeth for /d/ and /t/. MIs are then used to semi-automatically segment the audio/motor data found in a database of speech/motor trajectories recorded from 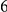 subjects.

Subsequently, a simple regression method (namely, a feed-forward neural network) is employed to build an Audio-Motor Map (AMM), which converts audio features of the isolated segment to features of the related MI. At an abstract level, the AMM is a mathematical proxy of a mirror structure [2], [3], reconstructing the distal speaker's *speech act* while listening to the related fragment of speech. According to a widely accepted account on the dorsal-ventral partitioning of the brain auditory system [4], [5] the AMM would be located in the dorsal stream, receiving input from the superior temporal gyrus (STG) projecting to the posterior parietal cortex and then to frontal regions (e.g., Broca's area) (note that the localization of the AMM in the brain does not necessarly imply a critical role of the AMM in speech perception, it might be critical for the speech learning phase only [5], [6]).

To test the approach, we devised three experiments involving a classifier in the form of a Support Vector Machine [7]. The main question is: can the use of MI-based features, either those recorded in the database (the “real” motor features) or the AMM-reconstructed ones (a more ecological scenario), improve the classifier's performance?

### Related Work

In the ASR community, the combination of explicit speech production knowledge and audio features has already been proposed (see, e.g., [8] for a review) as an alternative to the classic approach, in which speech production variability (e.g., due to speaking rate) and coarticulation (the phenomenon by which the phonetic realization of a phoneme is affected by its phonemic context) are directly and implicitly modeled in the acoustic domain. Here we restrict our investigation to the task of discriminating two bilabial from two dental consonants, so that we can lift a number of working assumptions and technical difficulties that have so far hampered a satisfactory integration of motor information into ASR systems.

Additionally, in previous work it is not possible to properly identify which aspects of the recognition process benefit from motor information. For example, motor knowledge may improve the modeling (and so the identification) of coarticulation effects that are seen in the training data set, but not necessarily improve the recognition of phonemes in unseen contexts, i.e., it may not necessarily improve the generalization ability of the ASR system. The experimental setup we have designed has the main goal of investigating whether and when motor information improves the generalization ability of a phoneme classifier.

It is known since the Sixties [9] that the audio signal of speech cannot be effectively segmented down to the level of the single phoneme, especially as far as stop consonants such as bilabial plosives are concerned; in particular, their representations in the audio domain are radically different according to the phoneme which immediately follows. It remains an open question then, how humans can distinctly perceive a common phoneme, e.g., /b/, in both /ba/ and /bi/, since they have access to the speaker's audio signal only. The explanation put forward by the Motor Theory of Speech Perception (MTS, [10]) is that, while perceiving sounds, humans reconstruct *phonetic gestures*, the physical acts that produce the phonemes, as they were trained since birth to associate articulatory gestures to the sounds they heard.

However, even ignoring the MTS, a very controversial theory indeed, recently reviewed and revised [11], [12], the use of speech production knowledge in speech recognition is appealing, in that the coupling of articulatory and audio streams allows for explicit models of the effects of speech production phenomena on the acoustic domain. In general, when the phonetic stream is directly mapped onto the acoustic dimension as in the standard approach to ASR, these effects cannot be precisely modeled, or cannot even be modeled at all. When exactly does /a/ affect the phonetic realization of /b/ in /ba/? What happens in the acoustic domain when /o/ is uttered with an exaggeratedly open jaw?

Different solutions have been proposed to integrate speech production knowledge into an ASR system and different types of speech production information have been used, ranging from articulatory measurements [13]–[15] to symbolic non-measured representations of articulatory gestures that “replicate” a symbolic phoneme into all its possible articulatory configurations [16], [17].

Although increased word recognition accuracy is sometimes reported when speech production knowledge is included in ASR, it is commonly held that the potential of speech production knowledge is far from being exhaustively exploited. Limits of current approaches include, e.g., the use of the phoneme as a basic unit (as opposed to articulatory configuration) which appears to be too coarse, especially in the context of spontaneous spoken speech, and the lack of a mechanism accounting for the different importance of articulators in the realization of a given phoneme (e.g., in the production of bilabials the lips are critical whereas the tongue is not).

As well, the traditional approach in which the speech signal is represented as a concatenation of phones (the “beads on a string” approach [18]) poses a number of problems to an accurate modeling of spontaneous speech, in which coarticulation phenomena such as phone deletion or assimilation (where a phone assimilates some articulatory gestures of the preceding/following phone), distorting the acoustic appearance of phonemes, are frequent and not always predictable. These problems call for finer-grained basic units. To partly compensate for such a limitation we propose an alternative approach where the audio signal is segmented using phone-specific articulatory patterns, expectedly more distinctive and stable than acoustic features.

During recognition, articulatory gestures have to be recovered from audio information as audio is the only signal available. Reconstruction of articulatory features has been attempted for a long time, but in most cases it is not derived from articulatory *data* gathered from human subjects. One pioneering case is that of Papcun et al. [19] where the AMM is carried out by a Multilayer Perceptron. Our procedure for building the AMM is deeply inspired by this work. The Multilayer Perceptron attempts the best recovery of all articulators giving equal importance to all of them; this could be, in general, problematic, since non-critical articulators will have high variance during the utterance of unrelated consonants [19], [20]. For example, the tongue position is expected to exhibit high variance while, e.g., velar plosives such as /k/ and /g/ are uttered. This is the main reason why an AMM is in general a one-to-many mapping: different articulatory configurations result in the same acoustic realization. Solutions to properly address the ill-posedness of the AMM have been proposed by Richmond et al. [21] and Toda et al. [22]; here we do not address the issue directly; rather, we consider two articulators only, therefore alleviating the problem.

Interestingly, the idea of using information about the mechanisms involved in the production of a human action to improve its classification/recognition (in a domain different from the production domain) has not only been applied in the context of speech recognition. For example Metta et al. [23] and Hinton [24] have shown that articulatory data can improve accuracy in automated hand action classification.

## Materials and Methods

### Data Set

#### Subjects and Set-up

Six female Italian native speakers were recorded while uttering Italian words and pseudo-words. Words were mainly stress-initial, e.g., “matto”, “nome”, “strada” (mad, name, road), and were chosen in order to have consonants both at the beginning and in the middle of words, followed by different vowels and consonants. The data recording setup included a *Laryngograph Microprocessor* device (Laryngograph Ltd., London, www.laryngograph.com) which gathers a speech audio signal and an electroglottographic (EGG) signal at 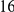 KHz sampling rate; and an AG500 electromagnetic articulograph (Carstens Medizinelektronik GmbH, Germany, www.articulograph.de) that records the 3D positions of a set of sensors glued on the tongue, lips and front teeth during speech production at a sampling rate of 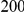 Hz. A full description of the acquisition set-up and the obtained database can be found in [25].

The subset used in this work comprises the 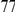 words in the database which contain /b/, /p/, /d/ or /t/. This includes utterings from each of the 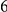 subjects; consonants are found both at the beginning of the word or in the middle; and they are followed by either /a/,/e/,/i/,/o/,/u/,/r/ or /s/.

#### MI-Based Signal Segmentation

**Figure 1:**
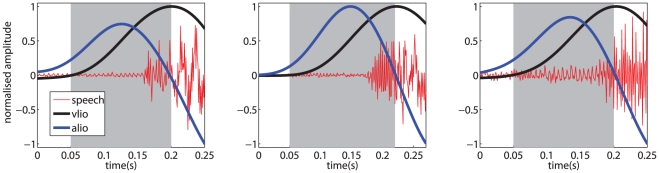
Speech signal and motor trajectories. The speech signal and motor trajectories (smoothed using a moving average filter) of lips opening velocity (vlio) and acceleration (alio) during utterances containing /b/. Left to right: /ba/, subject 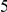; /ba/, subject 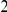; and /bufalo/, subject 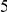. The gray zone denotes the detected start and ending of the plosion. All signals are normalized over the indicated time frame, for visualization purposes.

We define the length of a phone in terms of the MI underlying its production; the audio signal is, therefore, segmented according to it. A qualitative examination of the synchronized audio and motor signals obtained from utterances of /b/, /p/, /d/ and /t/ by different speakers indicates that common patterns can actually be found in the behavior of the related articulators. For instance, as is apparent from Figure 1, recurring shapes of the lips opening velocity and acceleration appear when both /ba/ and /bufalo/ are considered, even when uttered by different speakers. The same patterns can be observed and are qualitatively clear when other words containing /b/ and /p/ are considered, both when the phoneme appears at the beginning or inside a word, and regardless of the coarticulating phoneme.

These observations visually confirm the basic taxonomy of stop consonants as found in any linguistics textbook. In particular, all considered consonants are plosives, i.e., consonants that involve a complete blockage of the oral cavity followed by a fast release of air. /b/ and /p/ are bilabials (blockage produced using the upper and lower lips) while /d/ and /t/ are dentals (blockage produced using the tongue tip and the upper teeth). The following motor invariants are then defined and associated with the consonants under examination:

- Let 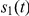 and 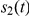 be the signals associated with sensors placed on two phonetic actuators (e.g., the upper and lower lips), and 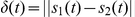 be their Euclidean distance. Then, a plosion is defined as the interval between two instants 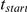 and 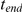 such that 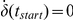 and 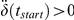, and 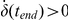 and 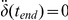.
- For /b/ and /p/, the sensors on the upper and lower lip are considered for 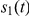 and 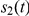, whereas for /d/ and /t/ those on the tongue tip and upper teeth are. In turn, the associated distances will be denoted as lio (lips opening) and ttu (tongue tip - upper teeth distance). As well, the respective velocities and accelerations will be denoted by vlio, vttu, alio, attu.

The first condition physically defines a plosion, e.g., considering lio, 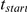 marks the onset of the act of opening the lips (null velocity, positive acceleration) while 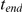 is found at the instant of maximum opening velocity and zero acceleration. The choice of cutting the signals at 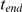 rather than, say, when the lips are still and lio is maximum is motivated by the need to capture the plosion only, with as little as possible of the following phone. By manual (audio) inspection of the audio segments so obtained, we could actually verify that only a tiny fraction of the coarticulating phone could be heard at the end of the uttering.

The second condition then selects an appropriate pair of articulators needed for the phoneme under consideration. This schema matches the above-mentioned taxonomy. In Figure 1 the gray zone indicates the detected interval of time using conditions 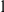 and 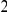. We expect that the same schema could be used to identify relevant MIs for other consonants, e.g., a velar plosion for /k/ and /g/ and so on – of course, suitable sensors must have been in place in that case.

The segmentation is carried out semi-automatically: for each utterance, all sequences matching the above conditions are displayed and the associated speech is played, so that the experimenter can choose whether the sequence is a correct guess or it is a false positive. In this experiment we only monitor lio and ttu, so that false positives appear, e.g., when considering /ts/ and /dz/. This is why, at this stage, a completely automatic segmentation cannot be enforced. If the sequence is accepted, it is labeled with the associated consonant, the speaker, and the coarticulating phoneme. For example, from the word /bronzo/ (bronze) a /b/ sequence is extracted, and the letter “r” is stored as the coarticulating phoneme. This way, from the original 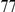 words and pseudowords, a total of 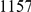 audio/motor sequences are extracted, with a length of 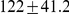 milliseconds (mean 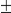 one standard deviation), minimum length 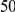 milliseconds, maximum length 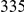 milliseconds.

### Training the Audio-Motor-Map

The procedure for building the AMM closely follows that outlined in previous literature [19], [21], [26] where a multi-layer perceptron neural network was employed to reconstruct articulators' positions from an audio stream. More in detail, the speech spectrogram was there used to predict, instant by instant, the position of the articulators of interest. Here we apply a similar approach to reconstruct the velocity and accelerations of lio and ttu, in order to avoid as much as possible taking into account physical differences among subjects (e.g., the width of the mouth, etc.).

For each of the 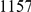 audio sequences, the spectrogram is evaluated over 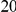-milliseconds long Hamming windows (slices), using a 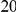-filter Mel-scale filterbank between 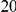 Hz and 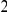 KHz. Each slice overlaps by 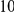 milliseconds with the preceding slice. Each single sample of vlio, alio, vttu and attu is then associated to 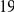 surrounding spectrogram slices, covering about 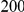 milliseconds of speech and centered around the sample itself. With this “sliding spectrogram window” method, the four trajectories are completely reconstructed. The Mel filters, the spectrogram and (later on) the cepstral coefficients of the audio signal are extracted using the off-the-shelf speech recognition Matlab package *Voicebox* [27].

**Figure 2:**
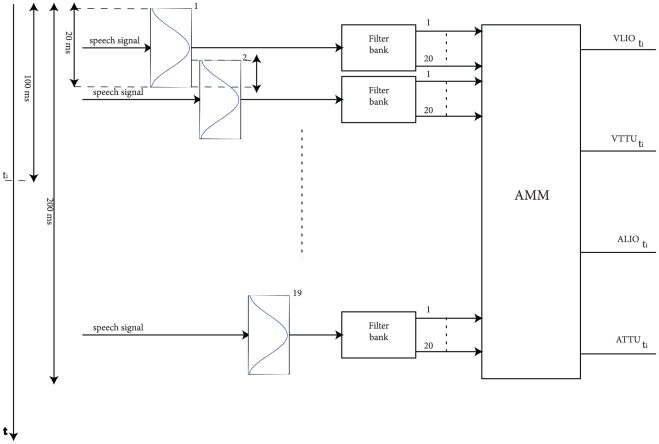
From speech signal to reconstructed motor information. The AMM is first trained on training speech data and then used, during testing, to reconstruct motor information from the testing speech data. To reconstruct a single sample of vlio, alio, vttu and attu at time 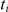 the spectrogram of nineteen 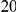-millisecond long Hamming windows is evaluated. One window is centered at time 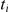, 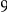 windows precede it and 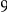 windows follow it. Each window overlaps by 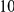 milliseconds with the preceding window. The spectrogram is computed by using a 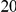-filter Mel-scale filterbank.

About 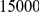 samples are extracted from the original 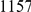 audio/motor sequences; each input sample consists of 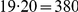 real numbers, while the output space is given by the 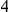 trajectory points of the motor signals (see Figure 2). A feed-forward neural network is set up in order to build the AMM, with 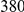 input units, one hidden layer with 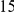 units and 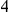 output units; the net is trained via the Scaled Conjugate Gradient Descent method [28] and the activation is a logistic sigmoidal function.

Training is done via early stopping on the appropriate validation set (see the “Evaluation setting” section for details). This procedure is repeated over 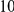 random restarts, and then the network with best average performance over the 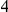 output dimensions is stored. The performance measure is Matlab's embedded mean-square-error with regularization function, in which after some initial experiments we set the regularization parameter at 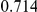. This value, as well as all other parameters, have been found in an initial experimentation phase, by slightly altering values suggested in literature and/or in the Matlab manual.

No sample normalization is performed, in order to preserve the time structure of the spectrogram windows. Targets are normalized in order to lie within the range 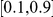, since the logistic activation function has asymptotic values of 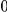 and 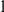.

### Phone classifiers

The phone classifiers are binary classifiers, the two classes are bilabial (/b/ and /p/) and dental (/d/ and /t/) plosive consonants.

#### Feature sets

Four different feature sets (one per each phone classifier) were compared.

“Audio” is a set of 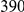 cepstral coefficients extracted from the audio signal as follows. We consider a set of 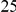-milliseconds long “time slices” of the signal. From each slice 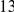 cepstral coefficients, plus their first- and second-order derivatives, are evaluated using a Mel-scale filterbank comprising 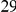 filters in the bandwidth from 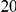 Hz to 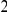 KHz; this results in 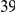 coefficients for each slice. This is a state-of-the-art set of features according to recent literature [29], [30] in which the single slices are classified as belonging to a phoneme or another with a certain probability, and then a time-sequence probabilistic method (typically, a Hidden Markov Model) is used. In our case, a whole variable-length sequence must be classified, so we consider 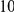 slices uniformly distributed across the sequence itself in order to cover it completely. In case the sequence is shorter than 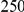 milliseconds, the slices are allowed to overlap, whereas in the opposite case there are gaps between them.

“Real motor” is a set of 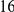 coefficients evaluated as follows: for each signal considered (vlio, alio, vttu and attu), a least-squares piecewise Hermite cubic interpolation is generated over the sequence. This results in 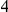 real numbers per signal (constant, I-, II- and III-order coefficient of the cubic interpolant). The choice of interpolating the signal trajectories is motivated by the need to capture the qualitative (plosive, in this case) behavior of the sensors abstracting away from, e.g., the length of the plosion, and to compactly represent it. Preliminary manual inspection of the trajectories has convinced us that a cubic fit would adequately capture their shapes.

“Reconstructed motor” refers to the same procedure as above, but applied to the AMM-reconstructed signal curves.

Lastly, “Joint” denotes a decision procedure obtained by averaging out the label probabilities obtained from the best classifiers for the audio and reconstructed motor features, and then using a threshold at 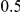.

#### Support Vector Machine-based classifiers

The classifiers are all based on a Support Vector Machine [7] with Gaussian kernel and hyperparameters 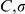 found by grid-search. Samples are normalized by subtracting the mean values and dividing by the standard deviations, dimension-wise, in the real motor and reconstructed motor cases, while no normalization is applied to the audio features. The off-the-shelf SVM package *libsvm* [31] has been used.

Support Vector Machines output decisions but not the probabilities of their decisions, i.e., the posterior probabilities. Only approximate estimations of the posterior probabilities can be computed. The *libsvm* implementation provides these estimations that are necessary for the “Joint” feature set based classifier.

## Results

We first describe the evaluation setting and then show the performance of the AMM and the accuracy of several phone classifiers in three experimental scenarios.

### Evaluation setting

As is standard practice in machine learning, the obtained dataset was divided into splits to perform cross-validation (CV). Six CV schemas were devised in order to assess the overall accuracy of the phone classifier and its sensitivity to the factors causing speech variability (e.g., coarticulation). The 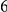 CV schemas are the following:

- *overall* The dataset is divided into 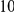 equally sized random disjoint sets. For each *split* (i.e., training/testing set pair) the training set contains 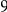 of these sets and the testing set contains the remaining set.
- *spk5vs1* The training sets contain samples uttered by 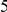 speakers while the testing set is uttered by the remaining speaker; this gives us 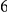 splits.
- *spk3vs3* Likewise, but training on 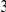 speakers and testing on the other 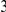. This results in 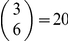 splits.
- *spk1vs5* Likewise, but training on 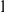 speaker and testing on the other 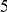, resulting in 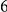 splits.
- *coart4vs1* The training sets contain samples with 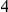 coarticulating vowels (i.e., vowels that follow the plosive), whereas the testing sets contain samples with the remaining two, plus /r/ and /s/. This gives us 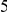 splits.
- *coart3vs2* Likewise, but training on 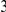 coarticulating vowels and testing on the remaining 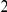 plus /r/ and /s/. This gives us 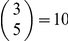 splits.

### AMM evaluation

**Figure 3:**
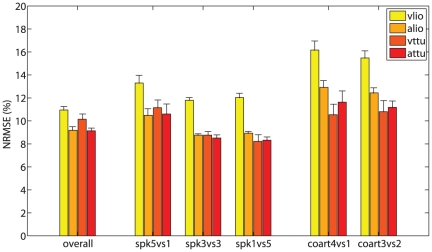
Quantitative performance of the AMM. For each cross-validation schema (overall, etc.) and output signal (vlio, etc.) the NRMSE average value and standard error of the mean are reported.

Figure 3 shows a quantitative assessment of the performance of the AMM. The measure of performance is the NRMSE (Normalized Root Mean Square Error), where the normalisation is over the range of each testing data set. The NRMSE ranges from 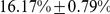 (vlio, *coart4vs1*) to 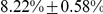 (vttu, *spk1vs5*). Regression upon vlio shows the largest error overall. Moreover, the error is on average larger for the per-coarticulation CV schemas.

**Figure 4:**
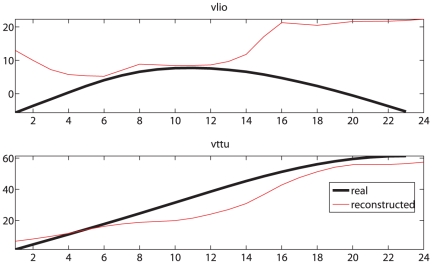
Real and AMM-reconstructed motor features. Real and AMM-reconstructed vlio and vttu for subject 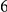 uttering the /t/ in *accento?* (accent). Notice the apparent gap in the quality of the reconstruction, favoring in this case the labiodental trajectory (vttu).

Although these figures do not really indicate whether AMM-reconstructed MIs will be effective in phoneme discrimination, they show that the error rate in regression has limited magnitude and does not differ dramatically across CV schemas and output signals. Qualitative inspection of the results (one example is given in Figure 4) shows that the AMM-reconstructed motor signals are on average rather similar to the real ones, at least as far as the range of values is concerned.

**Figure 5:**
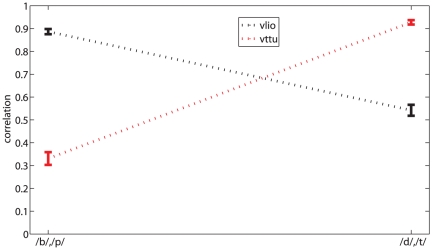
Double dissociation. Double dissociation of correlation between real and AMM-reconstructed MI (mean and standard error of the mean). Mean coefficients are significantly higher for vlio when “listening” to labials than dentals and vice-versa. The *overall* CV schema is used.

A definite trend is apparent, favoring the reconstruction of vlio over vttu when bilabials are presented to the AMM and vice-versa; the trend is numerically confirmed by checking the Pearson correlation coefficient between AMM-reconstructed and real MIs according to whether labials (/b/,/p/) or dentals (/d/,/t/) are presented as input to the AMM. As one can see in Figure 5, when the *overall* CV schema is used, a “double dissociation” pattern appears when comparing the correlation coefficients of vlio and vttu AMM-reconstructed from labials or dentals (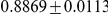 versus 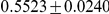 with Student's t-test 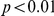 for vlio, and 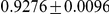 versus 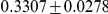, 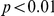, for vttu). In other words, when the AMM “hears” /b/ or /p/, it effectively reconstructs the trajectory of the lips, but less reliably that of the tongue tip; and dually, it reconstructs better the latter one when presented with /d/ or /t/. This pattern is repeated to an almost uniform extent when the other CV schemas are used, and also when alio and attu are checked.

### Phoneme discrimination

Each classifier uses one of the following feature sets: “Audio”, “Real Motor”, “Reconstructed Motor” and “Joint”.

#### Experiment 1

**Figure 6:**
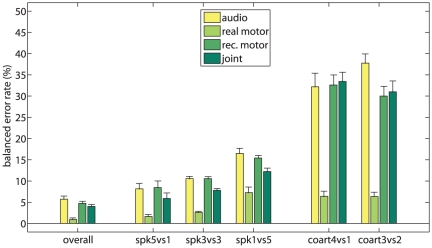
Results of experiment 1 and 2. Balanced error rate in classification of bilabials and dentals for each CV schema.

In the first experiment the performance of the phone classifiers is evaluated according to the *overall* CV schema using four different sets of features as input. Figure 6 (leftmost column) shows the results. The balanced error rate is shown as a comparative measure of performance. (This error rate is defined in our case as the average of the ratios of correctly predicted bilabials and dentals. With respect to the more popular standard error rate, i.e., the overall ratio of correctly guessed labels, it has the advantage of favoring models that can correctly guess *both* the bilabials and the dentals.)

The error rates obtained are, in turn, 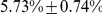 (mean 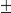 one standard error of the mean), 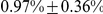, 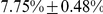 and 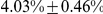. Student's two-tailed t-test shows 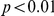 between real motor features and all the others, while in all other cases 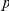 denotes weak statistical difference (e.g., 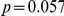 between audio and joint features). Together with the error rate values, this lets us claim that there is a marginal advantage in using joint features over audio only, but that a large and evident advantage is found using the real motor features over all the others.

#### Experiment 2

Experiment 2 replicates Experiment 1 using the remaining CV schemas. Figure 6 (from column *spk5vs1* to column *coart3vs2*) shows the results. Consider the per-speaker schemas, i.e., *spk5vs1*, *spk3vs3* and *spk1vs5*. The real motor features are, again, strikingly (and significantly, 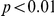) better than all others, with increasing error rates of 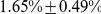, 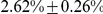 and 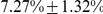 for *spk5vs1*, *spk3vs3* and *spk1vs5* in turn. Increasing (and larger) error rates are found when using audio and reconstructed motor features in all schemas, with no significant statistical difference. Significantly different performances are obtained with the joint features in the *spk3vs3* and *spk1vs5* schemas (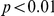 with error rates, in turn, of 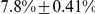 and 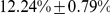).

In the per-coarticulation cases, the error rate is generally high (between 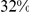 and 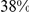 where chance level is 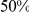). It is statistically similar (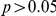) among audio, reconstructed motor and joint features in the *coart4vs1* schema, whereas in the *coart3vs2* schema there are significant differences (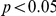) between audio and joint features, and audio and reconstructed motor features. The real motor features, again, perform dramatically better (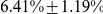 and 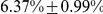 for *coart4vs1* and *coart3vs2* respectively).

In general, it is when the classification task becomes more difficult (i.e., decreased speech variability in the training data and increased speech variability in the testing data) that the reconstructed motor features lead to significant improvements, either when combined with the audio features (as in the *spk3vs3* and *spk1vs5* schemas) or alone (as in the *coart3vs2* schema).

#### Experiment 3

**Figure 7:**
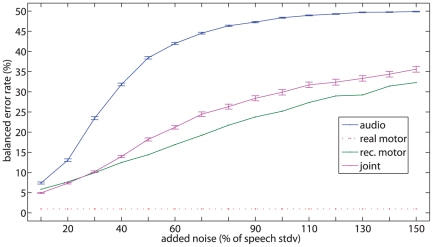
Results of experiment 3. Balanced error rate in classification of bilabials and dentals for the *overall* CV schema as noise is added.

Lastly, in Experiment 3 the comparison among feature sets is evaluated with the *overall* CV schema (which gives the best results in Experiment 2), as white noise is added to the audio signal. The intensity of noise is changed from 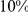 to 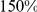 of the standard deviation of each utterance considered; for each sequence, 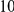 noisy ones are generated, in order to obtain a larger statistical basis. Figure 7 shows the results.

The real motor features, not affected by noise, are shown as comparison, and stay at the above mentioned error rate (see Experiment 1) of 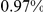. The error rate of the other sets of features, when noise is at 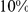, is only slightly worse than that of Experiment 1 (the same case with no noise): namely, 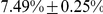, 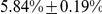 and 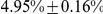 for audio, reconstructed motor and joint features in turn. As the level of noise is increased though, the audio features' error rate increases superlinearly until it reaches about 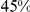 when the noise is at a 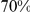 level, going then asymptotically to chance level. As opposed to that, the reconstructed motor features exhibit a much higher resilience to noise, increasing the error rate only linearly and reaching, e.g., 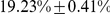 when the noise is at 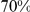. At the maximum level of noise, 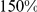, the reconstructed motor features still keep the error rate at 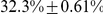 while the audio features essentially reach chance level. Actually, we ourselves checked how some of the phones sound when the noise is so high, and found them very hard to understand.

Lastly, the joint features perform better (or as well as) the reconstructed motor features at low levels of noise (until 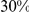), while they then become less useful than the reconstructed motor alone. This is obviously due to the weak performance of the audio features. The t-test reveals statistically different mean error rates (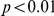) for all levels of noise, except for reconstructed motor and joint when the noise is at 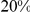 and 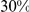.

## Discussion

### Do Motor Features Help?

The experimental results presented in the previous section clearly prove that, at least in the cases examined, and with the set of employed machine learning techniques, the answer to the question posed in the introduction, “can the use of MI-based features improve a phoneme classifier's performance?” is “yes”.

Overall, 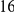 features extracted from motor invariants detected with an articulograph (what we have called real motor features) exhibit dramatically better error rates than 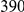 state-of-the-art audio features in an automated discrimination task between two bilabial and two dental consonants. Since the discrimination is performed using an absolutely standard classifier (and, according to the literature, a good one), that is, a Support Vector Machine with Gaussian kernel whose hyperparameters are found via grid-search, this result should have a somehow more general validity than what is shown in this paper.

The performance gap is apparent and statistically significant in all our experiments. It increases as the training sets are restricted, for example when per-subject (i.e., training on some subjects, testing on the others) or per-coarticulation (i.e., training on some coarticulating phonemes and testing on others) tests are conducted. This clearly indicates that MI-based features are somehow “more invariant” than audio-based ones across subjects and coarticulation – a quantitative confirmation of a basic intuition, almost common-sensical: to produce, e.g., a bilabial, the act of the labial plosion is common to all human beings and is not affected by coarticulation. This is one more hint at the fact that the use of motor features could be a great leap forward in ASR.

Now obviously, knowing that motor information is useful to improve ASR is just half of the story, since the problem of *gathering it* during speech recognition is still unexplored – one cannot expect the standard user of an ASR system to wear an articulograph while, e.g., dictating. Here the MTS and the theory of mirror neurons inspire us to build an AMM, that is, to try and reconstruct the distal speech acts from the audio signal alone. All in all, not even humans have access to the distal speaker's motor data, and recent studies, among which D'Ausilio et al.'s [1], indicate that they might be reconstructing it while hearing the sound of speech; and that this mechanism is activated mainly in hostile conditions (e.g., in the presence of noise).

Our Audio-Motor-Map, this one too built using a standard machine learning method (namely, a feed-forward neural network), is able to reconstruct the MIs to such a degree of precision that the same 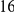 motor features, extracted from these reconstructed trajectories, exhibit comparable or better error rates than those found with the audio features when the training sets are restricted (Experiments 1 and 2); and they boost a largely and significantly *better* performance than the audio ones, as noise is added to the audio signal (Experiment 3). This latter result seems to be somehow in agreement with what D'Ausilio et al. have found using TMS on humans.

Note that in the most critical cases (i.e., when the training data sets are extremely restricted) of Experiments 1 and 2 the reconstructed motor features outperform the audio features. These results and the results of Experiment 3 suggest that when the difficulty of the classification task increases (because of an increased ratio between speech variability in the testing data and speech variability in the training data) the reconstructed motor features become more and more useful for the task.

Lastly, when audio and reconstructed motor features are joined using a simple probabilistic schema, the error rates are sometimes significantly better than when the feature sets are used independently. When one set of features is obviously far worse than the other, such a joint model performs in-between (e.g., consider Experiment 3 when noise is higher than 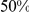); a more interesting case is that found in Experiment 2, CV schemas *spk3vs3* and *spk1vs5*, where no clear advantage is seen when using either the audio or the reconstructed motor features alone, while the joint models perform significantly better. This means that the MI-based models are correctly classifying with high probability some consonants that the audio-based models moderately misclassify; and vice-versa. Sometimes the audio features help, sometimes the MI-based features do.

This indicates that motor features, even when the audio signal is the only source of information available (a realistic scenario) can improve the discrimination of phonemes.

### Further Remarks

The experiments presented in this paper are inspired by the intuition that the proficiency of humans in speech recognition is grounded in the interaction between production and understanding of speech in the human brain. Alvin Liberman's motor theory of speech perception, although controversial and recently reviewed and revised [9]–[12], provides a theoretical framework to this intuition, which recent neurological evidence [1] supports even further; our findings seem to support the claim of MTS, but clearly more experiments are required, with larger data sets, e.g., more words, more subjects and more sensors.

In this work, also a novel way of segmenting the speech signal is introduced. The traditional equal-length segmentation, carried out using acoustic properties only, has strong limitations mainly due to intra-speaker speech variability and to coarticulation. Here we propose to segment the audio signal using the articulators' trajectories to detect the beginning and end of phonemes. The choice of the articulators and the conditions on the trajectories are established according to basic rules of the phonetic production; for example, /b/s are identified using the beginning and end of a bilabial plosion. With respect to the traditional speech segmentation, this approach focuses on the *act* that produced the sound. To capture this act, we use the coefficients of a cubic fit of the motor trajectories, so to obtain a qualitative representation of it.

About the AMM: from an information-theoretical point of view, AMM-reconstructed motor features do not carry more information than what already is in the speech signal. The AMM is a function, so one could see this technique as “just a better way of extracting ASR features from speech”. The main advantage in using it is that it is highly bio-inspired, having been trained to associated human speech data to motor data. The double dissociation observed (see Figure 5) reflects the rather predictable phenomenon that consonant-critical articulators exhibit less variance than non-critical ones (e.g., when a /b/ uttered the labial trajectory is highly constrained, as opposed to the tongue-dental trajectory). This results in a better prediction of bilabial (dental) trajectories when the AMM is presented with a bilabial (dental) consonant.

Lastly, notice that in Experiment 2 the AMM-reconstructed motor features perform, in general, as well as the audio features, while the real motor features are by far better. So, at first sight, one could be tempted to think that a better reconstruction should achieve better error rates, getting close to those of the real motor features; but on the other hand, the AMM uses the speech signal too, so it is not clear whether a much better reconstruction can be achieved in our case at all. A larger training database and more extensive experiments could shed light on this still open point.
